# Very small deletions within the *NESP55* gene in pseudohypoparathyroidism type 1b

**DOI:** 10.1038/ejhg.2014.133

**Published:** 2014-07-09

**Authors:** Faisal I Rezwan, Rebecca L Poole, Trine Prescott, Joanna M Walker, I Karen Temple, Deborah JG Mackay

**Affiliations:** 1 Human Genetics and Genomic medicine, Faculty of Medicine, University of Southampton, Southampton, UK; 2 Wessex Regional Genetics Laboratory, Salisbury District Hospital, Salisbury NHS Foundation Trust, Salisbury, UK; 3 Department of Medical Genetics, Oslo University Hospital, Oslo, Norway; 4 Queen Alexandra Hospital, Portsmouth, UK; 5 Wessex Clinical Genetics Service, Princess Anne Hospital, University Hospital Southampton NHS Foundation Trust, Southampton, UK

## Abstract

Pseudohypoparathyroidism (PHP) is caused by reduced expression of genes within the *GNAS* cluster, resulting in parathormone resistance. The cluster contains multiple imprinted transcripts, including the stimulatory G protein α subunit (Gs-α) and *NESP55* transcript preferentially expressed from the maternal allele, and the paternally expressed XLas, A/B and antisense transcripts. PHP1b can be caused by loss of imprinting affecting *GNAS A/B* alone (associated with *STX16* deletion), or the entire *GNAS* cluster (associated with deletions of *NESP55* in a minority of cases). We performed targeted genomic next-generation sequencing (NGS) of the *GNAS* cluster to seek variants and indels underlying PHP1b. Seven patients were sequenced by hybridisation-based capture and fourteen more by long-range PCR and transposon-mediated insertion and sequencing. A bioinformatic pipeline was developed for variant and indel detection. In one family with two affected siblings, and in a second family with a single affected individual, we detected maternally inherited deletions of 40 and 33 bp, respectively, within the deletion previously reported in rare families with PHP1b. All three affected individuals presented with atypically severe PHP1b; interestingly, the unaffected mother in one family had the detected deletion on her maternally inherited allele. Targeted NGS can reveal sequence changes undetectable by current diagnostic methods. Identification of genetic mutations underlying epigenetic changes can facilitate accurate diagnosis and counselling, and potentially highlight genetic elements critical for normal imprint setting.

## Introduction

Pseudohypoparathyroidism (PHP) is caused by insensitivity to parathormone (PTH), and the majority of cases are associated with insufficiency of the G protein stimulatory alpha subunit (Gs-α).^1^ In PHP type 1a (OMIM #103580), additional features may include obesity, short stature, brachydactyly, ectopic ossification and cognitive impairment. PHP1b (OMIM #603233) is classically defined as sharing the biochemical but not the skeletal features of PHP1a, though this distinction is not absolute.^2^ PHP1b is not associated with coding mutations of *GNAS*, but with epigenetic errors altering its expression. The *GNAS* gene cluster (OMIM +139320) on human chromosome 20 is under complex imprinted regulation, with transcripts variably expressed from the maternal allele, the paternal allele, or biallelically, in a tissue-dependent fashion.^3^ The clinical suspicion of PHP1b is confirmed by detecting abnormal DNA methylation at imprinting control regions within the *GNAS* cluster.^4, 5, 6^ Some patients show alterations at multiple imprinting control regions (ICRs) within the cluster, but the most consistent aberration is hypomethylation of the A/B differentially methylated region directly upstream of the major coding isoform of *GNAS*. Isolated hypomethylation of this region is the commonest alteration in familial PHP1b; in all cases so far described, it has been associated with deletion of a presumed regulatory element within the *STX16* gene.^7, 8^ Much rarer are deletions affecting *NESP55*;^9, 10, 11^ these are accompanied by epigenetic disruption throughout the locus, and evidence from murine models suggests they disrupt transcription and thereby primary imprint setting throughout the GNAS cluster.^12^ However, apart from these and some cases of paternal uniparental disomy (UPD20pat),^13^ the majority of PHP1b cases seem to be caused by a primary epimutation without an underlying genetic aberration.

We hypothesised that additional genetic variants may result in epimutations of the *GNAS* differentially methylated regions and screened PHP1b patients, using genomic next-generation sequencing through the *GNAS* cluster.

## Materials and methods

### Patient recruitment: ethics

Clinical diagnosis of PHP1b was made locally by referring clinicians, and patients were consented into the research study ‘Imprinting disorders–finding out why' (IDFOW: Southampton and South West Hampshire Research Ethics approval 07/H0502/85) through the UK Comprehensive Local Research network (www.southampton.ac.uk/geneticimprinting/informationpatients/imprintingfindingoutwhy.page, accessed September 2013). Diagnosis was confirmed by methylation-specific PCR and microsatellite analysis as described.^14^ Twenty patients had PHP1b with methylation anomalies affecting the *GNAS AB*, *XLAS*, *NESP-AS* and *NESP* differentially methylated regions (DMRs), but no evidence of UPD20 or *NESP* deletion;^9^ *STX16* deletions were also excluded; and there was no evidence of hypomethylation at any other imprinted locus. Two control PHP1b patients were included: one with UPD20pat and one with a deletion of *STX16*.

Clinical findings in the two families with genetic variants are presented in Table 1. Briefly:

Family 1: a non-dysmorphic sister and brother presented with exercise-induced muscle cramping in childhood and hypocalcemia. Skeletal survey was unremarkable. The mother was clinically unaffected.

**Figure 1:**
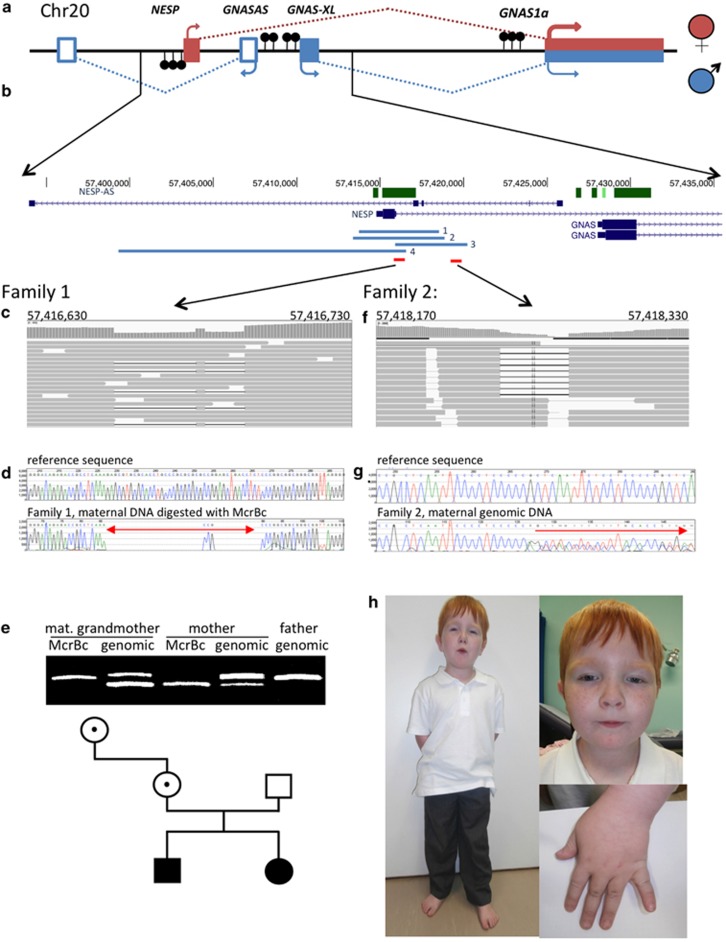
Identification of small GNASAS deletions in PHP1b patients. (**a**) Schematic of normal gene transcription and differential methylation within the GNAS cluster. Red and blue indicate maternal and paternal transcripts, respectively. Open and closed boxes represent non-coding and coding transcripts, respectively, while dotted lines indicate splicing. Black ‘lollipops' indicate locations of differential methylation, which is on the maternally derived allele for the *GNASAS*, *GNAS-XL* and *GNAS* A/B DMRs and the paternally derived allele for the *NESP55* DMR. (**b**) UCSC screenshot (GRCh37/hg19) of GNAS-imprinted region. Green boxes indicate CpG islands and blue rectangles linked by arrowed lines indicate positions of transcripts within the locus. Below the screenshot, blue lines illustrate deletions associated with PHP1b identified by (1, 2) Bastepe *et al*,^9^ (3) Chillambhi *et al*^10^ and (4) Richard *et al.*^11^ Red lines indicate positions of deletions in families 1 and 2. (**c**–**e**): the deletion in family 1. (**c**) Screenshot from IGV illustrating the deletion in case 1, family 1–note the black lines indicating deletion of nucleotides, and the reduction in overall sequence depth. (**d**) Sanger sequencing electropherogram of the same region in the mother of family 1, after restriction of genomic DNA with McrBc, which cleaves methylated DNA. Only the deleted allele remains, indicating that this deleted sequence is present on her paternally inherited allele. (**e**) Agarose gel electrophoresis of DNA from the parents and maternal grandmother in family 1, with and without restriction by McrBc to digest unmethylated DNA. This shows in the maternal grandmother that the deletion is digested, ie, present on the unmethylated paternal allele. In contrast, the deletion is on the maternal, unmethylated allele in the mother and absent from the unrelated father. (**f**–**h**) The deletion in case 9, family 8. (**f**) Screenshot from IGV illustrating the deletion in proband 2–note the black lines indicating deletion of nucleotides, and the reduction in overall sequence depth. (**g**) Sanger sequencing electropherogram of genomic DNA in proband 2, showing disruption of normal sequence originating at the deletion. (**h**) Clinical photographs of case 9. Note some facial features of PHP1a including flattening of nasal bridge, anteverted nares and small hands.

Family 2: a male presented at 2 years 3 months with faltering growth, increasing weight and global developmental delay, a consistently raised serum phosphate and subsequently a raised serum PTH. Skeletal survey was unremarkable. Family history was non-contributory (Figure 1h).

### DNA methylation analysis

Methylation status was initially determined by methylation-specific PCR, as described,^14^ and then more comprehensively assessed by methylation-specific pyrosequencing, as described^5^ in all patients, and compared with normal controls (Supplementary Table 1).

### Sequencing analysis

#### Targeted NGS

Genomic DNA was captured in the region chr20.GRCh37:g.57200800_57627000 using a targeted SureSelect array (Agilent Technologies UK Ltd, Wokingham, UK; 0.5–2.9 Mb capture). The array design (Agilent SureDesign, Agilent Technologies UK Ltd) had baseline × 2 coverage, moderate stringency, moderate masking of repeats and manual boosting in order to reduce replication of high GC-content baits. In total, 3 *μ*g DNA was sheared using a Bioruptor (Diagenode s.a., Seraing, Belgium) and then captured using the Agilent SureSelect system, according to the manufacturer's instructions. DNA samples were indexed, and 500 cycles of sequencing performed on an Illumina MiSeq (Reagent kit v2, Illumina UK, Chesterford, UK), generating ∼800 000 clusters, 89% passing filter and 7 Gb data. Further methodological details are available on request, and also the bed file of the capture array. Sequences were visualised on the Integrative Genomics Viewer v1.4 (www.broadinsitute.org/igv). Raw sequence data are available on request.

#### Long-range PCR and NGS

Genomic sequences: chr20.GRCh37:g.57413337_57418468 and chr20.GRCh37:g.57416569_57419096 (primers 5′-GCCCATCATTTGATTTTCTAGGGCCAAG-3′ 5′-GGAGCTGAGTACCAGTCTCTCAGGCAG-3′ 5132 bp and 5′-GCGCCAGTGCCTCCAGCTGCCG-3′ 5′-CCTTCCACACAGCTGCAGAAAATGAAG-3′ 2529 bp) were amplified in individuals with PHP1b. Sequencing libraries were prepared and indexed using the Nextera DNA sample preparation kit and Nextera indexing kit (Illumina UK) following the manufacturer's protocols, and sequenced using the MiSeq micro reagent kit, 500 cycles (Illumina UK).

#### Sanger sequencing

The deletions in family 1 (chr20.GRCh37:g.57416653_57416693del) and family 2 (chr20.GRCh37:g.57418256_57418290del) were amplified using primers 5′-GCGCCAGTGCCTCCAGCTGCCG-3′ 5′-CGTTCAACCCTGGTAGCCCGTAGGG-3′ (220 bp) and 5′-CCATGTTCACATGTAGCGAGGAGGG-3′ 5′-CGGGGGTTGGTATAGCTCTCAGTTGC-3′ (273 bp) and verified by Sanger sequencing. To determine inheritance in the mother and maternal grandmother, 200 ng genomic DNA was amplified after restriction digestion with restriction enzyme Mcrbc (New England Biolabs UK, Hitchin, UK) according to the manufacturer's instructions, as described.^15^

### Bioinformatic analysis

We developed a bioinformatic pipeline for identifying both single-nucleotide variants (SNVs) and copy number variants (CNVs). Sequence output from targeted NGS was aligned to a human reference sequence (UCSC Genome Browser hg19, http://hgdownload.cse.ucsc.edu/goldenpath/hg19/chromosomes/) using Burrows-Wheeler Aligner (BWA-MEM–version 0.7.5a) (http://bio-bwa.sourceforge.net) to produce BAM files (binary version of Sequence Alignment/Map Format). Duplicate reads were removed from the BAM files using Picard tools (version 1.95) (http://picard.sourceforge.net) to avoid biases that might skew variant predictions. Local indel realignment was undertaken by the ‘Indel Realigner' function from the Genome Analysis Toolkit (GATK–version 2.4–9^16^) to avoid misalignment that might cause mismatches and compromise base quality recalibration. Base quality recalibration was done using GATK ‘Table Recalibration' function, which analysed several covariates to remove systematic biases and improve accuracy of quality scores.^17^ All produced aligned files were further used to calculate average read depth in 1000 bps non-overlapping windows within the captured region (Supplementary Table 2). The BAM files were also used to normalise read depth between samples, which was used to compare samples between different pools (Supplementary Table 2). These realigned and recalibrated BAM files were then used to determine SNVs and CNVs. Variant (SNVs eg SNPs and indel alleles) predictions and genotyping were performed using the GATK ‘Unified Genotype' function for each sample by computing allele frequency distribution,^18^ and raw variant calls were outputted in VCF (variant call format). An individual variant filtration (such as Quality by Depth<2.0, Fisher Strand Bias>60.0 etc) was performed for both SNPs and indels using GATK Variant Filtration method to remove low quality and potentially false positive sites. Variant data were further annotated by Annovar to provide functional annotation of variants.^19^

Pindel (version 0.2.4^18^), a structural variant detection tool that can detect breakpoints of large deletions and medium-sized insertions from paired-end short reads, was used to perform CNV predictions. These CNVs were further filtered based on variant size, alternate allele frequency, CA/GT homopolymer repeats and known SNPs. These filters increased sensitivity and specificity of the predictions, which reduced false negatives and false positives.

The variants identified in Patients 1, 2 and 9 have been submitted to LOVD (www.lovd.nl/GNAS variants GNAS_00174, GNAS_00175, GNAS_00176).

## Results

In seven PHP1b patients, the *GNAS* locus (chr20.GRCh37:g.57200800_57627000) was enriched by targeted capture, and analysed by next-generation sequencing. On average, more than 98% of the captured regions had read depth greater than 10, showing that the sequence data were of high quality in our region of interest (Supplementary Table 3). Case 1 of family 1, one of two affected siblings, harboured a 40-bp deletion spanning the genomic co-ordinates chr20.GRCh37:g.57416653_57416693del (family 1, Figure 1c). This deletion was intronic to both *NESP55* and *NESP-AS*. It was not present in dbSNP137, Repeat Masker (http://genome.ucsc.edu) or in 168 control samples. The deletion was detected in the affected sibling and also in the siblings' mother who showed no clinical signs of PHP1b. To determine the parental origin of the deletion in the mother, her DNA was amplified after digestion with methylation-specific restriction enzymes *Bst*UI and *Mcr*Bc (which digest only unmethylated and methylated DNA, respectively). The deletion was detectable only in DNA restricted by *Mcr*Bc, demonstrating that it was present on her unmethylated, maternally inherited allele (Figure 1d and g). The maternal grandmother of the affected sibs also harboured the deletion, though methylation-specific restriction analysis in her case showed it to be on the paternally inherited allele (Figure 1g).

Building on this finding, the genomic region chr20.GRCh37:g.57413337_57419096 was amplified by long-range PCR and sequenced by NGS in samples from an additional 14 PHP1b patients with no known underlying genetic aberration. A 33-bp intronic deletion was identified (chr20.GRCh37:g.57418256_57418290del) in one further patient (case 9, Figure 1, Table 1). Targeted sequencing showed the same deletion to be present in his unaffected mother. The deletion was too distant from the DMR to use methylation-specific restriction to determine its origin in the mother. The deletion was not present in the maternal grandmother of patient 2 (data not shown); the maternal grandfather was deceased and no DNA was available for analysis, but he was not known to have any clinical features of PHP1b. The variant in family 2 represented a deletion of two of three copies of an 18-bp repeat element; deletion of one copy was represented within dbSNP137 as rs36230182 (minor allele frequency unknown); however, there was no evidence of any deletion in 168 normal controls. In the remaining 13 patients, CNVs were called by Pindel and SNPs were called by GATK, but no novel variants were identified (data not shown).

DNA methylation analysis showed the affected sibs in family 1 had essentially complete paternalisation of the *GNAS* cluster, while hypomethylation in proband 2 was incomplete, except at the GNAS A/B DMR (Supplementary Table 1).

## Discussion

Like other imprinting disorders, PHP1b is clinically and molecularly heterogeneous. Sporadic PHP1b is associated with UPD20pat and epimutation of DMRs throughout the *GNAS* cluster; most heritable PHP1b results from epimutation of the *GNAS A/B* DMR–associated with maternal *STX16* deletions–and rarely from deletions of *NESP55* of maternal origin. We used NGS of the *GNAS* locus to seek genetic lesions underlying epimutations in PHP1b patients. In two affected siblings, a variant was found in the first intron of *NESP55*, within the 3-kb deletion described by Bastepe *et al.*^9^ Further localised sequencing within this region revealed another deletion in a third, unrelated individual. Both variants were maternally transmitted, but the mothers showed no clinical or epigenetic evidence of PHP1b. In family 1, the clinically unaffected mother inherited the deletion from her own mother, showing that maternal inheritance of this deletion does not always cause PHP1b.

Large *NESP55* deletions are believed to abrogate germline transcription from the *NESP* promoter, resulting in aberrant methylation setting throughout the whole imprinted cluster.^9, 12^ Notably, the variants reported here are close to the transcriptional start site of *NESP55*, overlapping those described by Bastepe, Chillambhi and Richard *et al.*^9, 10, 11^ Such deletions potentially delineate critical sequences for imprint setting of this locus.

We detected variants in only two of 20 PHP1b index cases (albeit with different extents of sequencing). *NESP* deletions >1 kb were not detected in any individual. By contrast, *STX16* deletions are found in essentially all individuals with *GNAS A/B* hypomethylation. It remains unclear whether genetic *NESP* mutations are uncommon in PHP1b. It may be that in the patients we studied, some variants remained undetected because of discontinuities in sequence coverage; CNVs (like the *STX16* deletion) may arise between repetitive sequences, but these sequences cannot be unambiguously captured, results in reduced or absent coverage. Further variants may lie outside the region we targeted, in more distant chromatin-regulating domains. Alternatively, it may be that the majority of PHP1b is indeed sporadic, caused by epimutations without direct genetic cause. Genetic mutations underlying epimutations have been reported very infrequently in other imprinting disorders, including Russell–Silver Syndrome, transient neonatal diabetes mellitus and Beckwith–Wiedemann syndrome associated with KCNQ1OT1 (imprinting control region 2) hypomethylation. However, a significant minority of BWS patients with H19 (ICR1) hypermethylation have maternally inherited deletions or point mutations of ICR1.^20^ Genomic analysis of other patient groups could potentially identify mutations that would help to uncover further determinants of epigenetic function.

It is striking that the individuals with *NESP55* deletions were not identical, either in phenotype or in DNA methylation status. Clinical features in the affected individuals were somewhat atypical of PHP1b, which is associated chiefly with biochemical features of parathormone resistance in the absence of dysmorphism.^3^ The probands of family 1 have mild learning difficulties, and one has calcifications in brain tissue; the proband in family 2 has educational difficulties warranting educational support, and has short stature, which is more typical of PHP1a. It is recognised that a minority of individuals with PHP1b epimutations have clinical features overlapping with PHP1a.^21, 22, 23, 24^ In the three individuals with PHP1b, the deletions resulted in epimutations throughout the GNAS cluster; but while the siblings in family 1 had almost complete hypomethylation, the proband in family 2 had less marked hypomethylation, except at the GNAS A/B DMR. In family 1, a deletion was transmitted maternally through three generations, but only the two-third-generation cases 1 and 2 presented with PHP1b. This may be because the deletion is coincidental to PHP–though given the presence of deletions in two of 20 pedigrees, and their absence from controls and databases, this seems unlikely. It may be that these small deletions are not fully penetrant, and do not always cause PHP1b. Alternatively, the severity of the deletion's effect may increase with successive generations, causing apparent genetic anticipation–this phenomenon has been observed in maternally transmitted BWS associated with hypermethylation of H19.^15^ Investigation of further cases will shed light on these unresolved issues. In summary, genomic sequencing is a powerful tool with potential to reveal non-coding genetic variants that may underlie epigenetic disorders.

## Figures and Tables

**Table 1:** Clinical features in the affected individuals in families 1 and 2

	*Family 1*		*Family 2*
	*Case 1*	*Case 2*	*Case 9*
Current age	18 years	23 years	4 years
Pregnancy and delivery	Normal	Normal	Normal
Gender, birth weight and length, neonatal course	Female 3790 g, 51 cm at 41+3 weeks No neonatal problems	Male 3300 g, 49 cm at 41 weeks No neonatal problems	Male 2950 g at 40 weeks No neonatal problems
Family history	Healthy unrelated parents	Elder brother of case 1	Healthy unrelated parents
Presentation	Activity-induced aching in arms and legs, cramps in fingers at diagnosis at 12 years	Activity-induced aching in legs at 14 years, diagnosed with PHP1b at 17 years	Growth failure, rapid weight gain and global developmental delay by 2 years.
Early development	Walked at 17 months. Delayed early milestones	Walked at 19 months. No speech until age 4 years	Walked at 25 months. Speech and language delay noted by 3 ¾ years
Education and schooling	Attended special primary school. Currently a year behind in a normal school. Functions within the normal range cognitively, but has specific difficulties with short-term memory and writing/reading	Attended normal school with extra support. Lives independently. Pursuing post-secondary education. Above average cognitively, has specific learning difficulties, problems with short-term memory	Attends normal school with a statement of special educational needs
Examination	At 12 years 3 months: Height 155 cm (50th centile) Weight 47.7 kg (75th centile) OFC 53.3 cm (25–50th centile) Not dysmorphic	At 17 years 6 months: Height 173 cm (25–50th centile) Weight 57.5 kg (25th centile) OFC 53.3 cm (50–75th centile) Not dysmorphic	At 3 years 10 months: Height 97 cm (9–25th centile), weight 18.1 kg (75th–91st centile) OFC 53 cm (91st centile).
Skeletal and dental findings	Normal hands and feet. No subcutaneous calcifications. No dental problems	Normal hands and feet. No subcutaneous calcifications. Two incisors extracted to make space. Retained tooth in the maxilla repositioned surgically	Small 4th and 5th digits bilaterally. No subcutaneous calcifications
X-ray findings	Normal hands and feet	Normal left hand. Bilateral calcifications in subcortical white matter, basal ganglia, cerebellum	Normal hand X-rays. Normal bone density
Vision and hearing	Normal	Normal	Normal
Metabolic findings	At 12 years: Calcium 1.21 mmol/l (2.15–2.70) Albumin 45 g/l (36–48) Phosphate 2.69 mmol/l (1.20–1.80) Magnesium 0.82 mmol/l (0.71–0.94) Creatinine 46 *μ*mol/l (40–75) PTH 61.5 pmol (1.6–6.9)	At 17 years: Calcium 1.38 mmol/l (2.15–2.55) Albumin 44 g/l (36–48) Phosphate 1.80 mmol/l (0.7–1.50) Magnesium 0.71 mmol/l (0.71–0.94) Creatinine 58 *μ*mol/l (60–105) PTH 33.2 pmol/l (1.6–6.9)	At 2 years 3 months: Calcium 2.5 mmol/l; Phosphate 1.9 mmol/l (upper limit of normal 1.5) and PTH 3.3 pmol/l (normal <6.4). At 3 years: Calcium 2.48 mmol/l, Phosphate 1.9 mmol/l At 4 years: Calcium 2.43 mmol/l (2.15–2.6) Phosphate 1.88 mmol/l (0.9–1.6) PTH 10.9 pmol/l (0.9–9.9)
Other	Recurrent urinary tract infections. Thyroxine supplementation from age 14 years for borderline low free T4 with normal TSH	Age 19 years: Hyperechogenic foci in both kidneys, possibly normal variant	
GNAS sequencing	Exons 2–13: no coding variants	Exons 2–13: no coding variants	Exons 2–13: no coding variants

Abbreviation: OFC, occipito-frontal circumference; PTH, parathormone; TSH, thyroid stimulating hormone.
